# Exploring anxiety as a potential mediator of the association between home destruction and PTSD symptoms among hemodialysis patients in Gaza after 7th Oct 2023

**DOI:** 10.1371/journal.pmen.0000682

**Published:** 2026-08-20

**Authors:** Maha AbuZarifa, Yaser Hamam, Mohammed Hamam, Salah Wajdi Alaloul, Khalil Mostfa Alinaby, Mohammed Wajdi Alaloul, Yara M. Hijazi, Mohammed B. AbuZarifa, Rana AbuZarifa, Guido Veronese

**Affiliations:** 1 Al-Quds University, Al-Azhar Branch, Gaza, Palestine; 2 Faculty of Medicine, Al-Quds University, Jerusalem, Palestine; 3 Faculty of Medicine, Islamic University, Gaza, Palestine; 4 Faculty of Medicine, Al-Azhar University, Gaza, Palestine; 5 Faculty of Medicine, Ain Shams University, Cairo, Egypt; 6 Faculty of Pharmacy, Al-Azhar University, Gaza, Palestine; 7 Department of Human Sciences for Education "R. Massa", University of Milano-Bicocca, Milano-Bicocca, Italy; 8 Stellenbosch University, Department of Psychology, Stellenbosch, South Africa; PLOS: Public Library of Science, UNITED KINGDOM OF GREAT BRITAIN AND NORTHERN IRELAND

## Abstract

Home destruction is a severe traumatic event strongly associated with psychological distress, particularly in conflict settings. Hemodialysis patients face heightened vulnerability due to chronic illness and dependence on continuous medical care. This study examined whether anxiety mediates the relationship between home destruction and post-traumatic stress disorder (PTSD) among hemodialysis patients in Gaza following the military assault on October 7, 2023. A cross-sectional study was conducted across five dialysis centers in Gaza. Anxiety symptoms were measured using the Depression Anxiety Stress Scale (DASS-21), and PTSD symptoms were assessed using the PTSD Checklist for DSM-5 (PCL-5). Structural equation modeling with bootstrapped confidence intervals evaluated mediation effects. Among 383 participants, 83.6% reported moderate-to-extreme anxiety, and 58% met the PTSD cutoff. Home destruction was highly prevalent, affecting 66.1%, with 53.5% experiencing total loss of residence. Significant positive correlations were found among home destruction, anxiety, and PTSD. Mediation analysis showed that anxiety fully accounted for the association between home destruction and PTSD (significant indirect effect, non-significant direct effect after adjustment). In adjusted models controlling for gender and current residency, the total effect was no longer significant, consistent with full statistical mediation. The model explained 42% of the variance in PTSD scores, almost entirely through anxiety (R^2^ = 0.02 for anxiety from home destruction alone). Given the cross-sectional design, this mediation should be interpreted as a statistical association rather than evidence of a causal pathway, and longitudinal studies are needed to confirm temporal relationships. Although causality cannot be determined from this cross-sectional study, the observed indirect association suggests that integrating anxiety-focused mental health support into dialysis services may help reduce PTSD symptoms among conflict-affected hemodialysis patients. Pilot intervention studies are needed to test this hypothesis.

## Introduction

Humanitarian crises and armed conflicts are consistently associated with profound and enduring adverse effects on mental health, particularly among civilian populations exposed to repeated and cumulative traumatic stressors [1]. Beyond direct exposure to violence, armed conflicts disrupt fundamental aspects of civilian life through experiences such as home destruction, loss of loved ones, forced displacement, and chronic insecurity. These experiences are strongly associated with long-term psychiatric outcomes, including anxiety disorders, depression, and post-traumatic stress disorder (PTSD) [2–4]. Recent evidence from Gaza confirms that anxiety and depression fully mediate the relationship between trauma exposure and psychotic like experiences in the general population, further supporting the plausibility of anxiety as a mediator [5]. In Gaza, the situation is further compounded by catastrophic health system collapse; according to a recent UN report, approximately 40% of dialysis patients have died since October 2023 due to Israeli attacks on health facilities or lack of access to care [6].

Decades of political violence and military escalation in Gaza Strip have created a context of prolonged trauma exposure [7]. The last military assault, which began on October 7, 2023, has further intensified an environment marked by fear, bereavement, displacement, and uncertainty, contributing to widespread psychological distress and deterioration in mental health among civilians [2]. Epidemiological evidence from Gaza demonstrates alarmingly high prevalence of anxiety, depressive symptoms, and probable PTSD, reflecting the cumulative psychological toll of sustained exposure to war-related stressors [2,8,9]. For this study, home destruction is defined as the complete or partial physical demolition of a participant’s primary residence due to military action, resulting in forced displacement, loss of personal belongings, and disruption of social and healthcare access.

Among the most well-documented war-related stressors in the Palestinian context is home destruction [10]. Home demolition represents a particularly severe and destabilizing traumatic experience, as it simultaneously entails material loss, forced displacement, and persistent threats to personal and family security [10,11]. Empirical research indicates that individuals whose homes have been demolished exhibit significantly higher levels of psychological distress compared with those whose homes remain intact, including elevated symptoms of PTSD, anxiety, depression, and stress [12]. In Palestine, home destruction has been repeatedly identified as a critical trauma-related factor with lasting mental health consequences, extending beyond immediate material deprivation to encompass chronic feelings of helplessness, insecurity, and loss of control [10,12].

Hemodialysis patients represent a uniquely vulnerable subgroup within this context [13]. Their survival depends on regular access to medical care, stable transportation, electricity, water, and functioning health infrastructure—resources severely disrupted by conflict [14]. Wartime conditions have been associated with reduced quality of life, heightened psychological distress, and increased vulnerability to anxiety and trauma-related disorders among patients with chronic kidney disease living in conflict-affected settings [7,15]. Coupled with repeated exposure to war-related traumatic events, these stressors highlight the need to examine mental health mechanisms in this high-risk group.

A substantial body of research demonstrates a strong association between anxiety and PTSD. Anxiety frequently co-occurs with PTSD and plays a key role in the development and maintenance of PTSD symptoms [16–18]. Although PTSD is classified as a trauma- and stressor-related disorder with prominent anxiety features, substantial evidence indicates that heightened anxiety sensitivity is closely linked to greater PTSD symptom severity, supporting anxiety as a central mechanism in trauma-related psychopathology [18,19]. Given the substantial conceptual overlap between trauma-related anxiety and PTSD, anxiety cannot be assumed to be causally antecedent to PTSD [18]. Nevertheless, trauma theories suggest that exposure to prolonged environmental threat may generate heightened anxiety responses that co-occur with and potentially contribute to greater PTSD symptom severity [10,18,19]. Accordingly, anxiety was examined in the present study as a theoretically informed and exploratory explanatory pathway linking home destruction to PTSD symptoms [20,21].

Conceptualizing anxiety as a potential mediator requires careful consideration because anxiety and PTSD are closely related constructs [15,18]. PTSD is classified as a trauma- and stressor-related disorder and includes symptoms of hyperarousal, fear, and threat perception that overlap with anxiety [18]. However, previous research suggests that anxiety symptoms and anxiety sensitivity may represent important processes associated with the development and maintenance of PTSD symptoms in trauma-exposed populations [15–17]. In conflict settings, persistent structural adversities such as displacement, home destruction, and ongoing insecurity may first generate chronic anxiety regarding safety, survival, and access to essential resources [9,10,19]. Such anxiety responses may be associated with increased vulnerability to trauma-related psychopathology [15,16,18]. To our knowledge, no prior study has investigated whether anxiety mediates the relationship between home destruction and PTSD specifically among patients requiring life sustaining hemodialysis in a war zone. Therefore, the present study aimed to examine anxiety as a theoretically plausible, though exploratory, explanatory variable in the association between home destruction and PTSD symptoms.

### Primary objective

- To determine if anxiety fully or partially mediates the relationship between home destruction and PTSD symptoms among adult hemodialysis patients living in a conflict-affected setting. This study was not registered in a public trial registry, and no formal protocol was published. All analyses followed the prespecified plan described in the Methods.

### Secondary objectives

1. To assess the prevalence and severity of anxiety and PTSD symptoms in hemodialysis patients exposed to war-related stressors.
2. To examine the association between home destruction and anxiety levels.
3. To explore the association between anxiety and PTSD severity.
4. To evaluate whether the mediation effect persists after adjusting for demographic and displacement-related covariates (e.g., gender, current residency).

## Methodology

### Ethics statement

The study received ethical approval from the Research Ethics Committee of Al-Azhar University and was conducted in accordance with the ethical principles of the Declaration of Helsinki. All participants were adults (aged 18 years or older) and provided written informed consent before participation. Participation was voluntary, and participants were informed of the study objectives, procedures, potential risks, and their right to refuse participation or withdraw at any time without consequences for their medical care. No minors were included in the study; therefore, parental or guardian consent was not required. No financial compensation was provided. Interviewers were trained to monitor for signs of distress (e.g., crying, refusal to continue), and participants who became distressed were offered a break, the option to skip questions, or referral to the dialysis unit’s support staff (available at all five centers). Confidentiality was rigorously maintained throughout the study, and all responses were anonymized and securely stored to protect participant privacy.

### Study design and setting

This study adopted a cross-sectional design to investigate whether anxiety mediates the relationship between home destruction and PTSD among hemodialysis patients in Gaza following the war that began on October 7, 2023. Data were collected from five dialysis centers: Al-Shifa Hospital, Al-Aqsa Hospital, Nasser Medical Complex, Al-Rantisi Hospital, and MSF Belgian Field Hospital. Data collection was conducted between August 2025 and November 2025.

### Participants

The study population consisted of 383 adult patients undergoing maintenance hemodialysis. Participants were eligible if they were aged 18 years or older, had been receiving regular hemodialysis during the conflict period in Gaza, and were able to provide informed consent. Patients with severe cognitive impairment or inability to complete questionnaires were excluded. Recruitment occurred consecutively during routine dialysis sessions, and all participants provided informed consent. All participants lived in a high-intensity conflict zone with widespread exposure to events meeting DSM-5 Criterion A [22].

## Measures

Data collection was conducted through interviewer-administered questionnaires during dialysis sessions to ensure completeness and minimize missing data. Privacy and confidentiality were maintained throughout the process. The primary exposure variable, home destruction, was assessed using a single questionnaire item asking participants whether their original home was destroyed during the war. Responses were recorded in three points scale: (1) no destruction, (2) partial destruction, or (3) complete destruction. Partial destruction was defined as significant damage making the home uninhabitable but with some structure remaining; total destruction was defined as complete collapse or rendering of the home beyond repair. No objective verification (e.g., satellite imagery) was performed, as access was not possible during active conflict. Anxiety was measured using the Depression Anxiety Stress Scale (DASS-21) anxiety subscale. Scores were categorized as follows: normal (0–7), mild (8–9), moderate (10–14), severe (15–19), and extremely severe (≥20). PTSD symptoms were assessed using the PTSD Checklist for DSM-5 (PCL-5), with a cutoff score greater than 33 indicating clinically significant PTSD. Additional demographic and clinical covariates, including age, gender, marital status, education, income, dialysis duration, and comorbidities, were also recorded

### Statistical analysis

Data were first inspected for completeness and distributional properties. Continuous variables (e.g., DASS‑21 anxiety scores, PCL‑5 PTSD scores, dialysis duration) were summarized using means and standard deviations(SD). We assessed normality by histograms, Q–Q plots, and the Shapiro–Wilk test. Data with p < 0.05 was considered not normally distributed. Categorical variables (e.g., gender, marital status, education, type of residence damage, current residency) were described using counts and percentages.

Spearman’s rank correlations were used to examine associations among the primary exposure (home destruction severity), the mediator (anxiety score), and the outcossme (PTSD score). Additional bivariate tests explored relationships with demographic and displacement-related variables (S2 Table). After adjusting for multiple comparisons using the Holm method, only variables that remained significant were retained as covariates in the adjusted exploratory mediation model.

Although our mediation framework drew on the foundational Baron and Kenny (1986) approach, indirect effects were evaluated using bias-corrected bootstrap confidence intervals, consistent with current recommendations for mediation analysis [23]. Because cross-sectional mediation analyses cannot establish temporal ordering or causality, the present analysis was conducted to evaluate whether observed associations were consistent with the theoretically proposed role of anxiety as an intermediate variable linking home destruction and PTSD symptoms. Therefore, all findings are interpreted as statistical and associational rather than causal.

In this model, home‑destruction severity served as the independent variable, anxiety (DASS‑21) as the mediator, and PTSD severity (PCL‑5) as the outcome. Following the causal‑steps logic, mediation is supported when: (1) home destruction significantly predicts anxiety (path a); (2) home destruction significantly predicts PTSD (path c, total effect); (3) anxiety predicts PTSD while controlling for home destruction (path b); and (4) the direct effect of home destruction on PTSD is reduced or becomes non‑significant once anxiety is included (path c′). We estimated these relations using a structural equation modeling framework and sequential regressions examining the total effect (c), the a‑path, and the b and c′ paths. The indirect effect (a × b) was tested with 5,000 bootstrap resamples to obtain bias‑corrected 95% confidence intervals via the lavaan package in R; mediation was deemed present when the interval excluded zero. All bootstrap analyses used random seed 2025 for reproducibility. An adjusted exploratory mediation model was also estimated, controlling for gender and current residency. All analyses were performed using R software, with statistical significance set at p < 0.05.

## Results

### Participants characteristics

After a complete case analysis, three hundred eighty-three dialysis patients from five medical institutions were included in the final analysis. The majority were from Al-Shifa Hospital. The cohort was predominantly older adults (most aged ≥55), married, and had finished secondary school, with a slight female majority. Also, Socioeconomic vulnerability was marked: unemployment and low household income were common. Clinically, multimorbidity was prevalent (HTN/DM/heart disease), with long dialysis exposure frequently exceeding five years, limited transplant candidacy, and an inactive waiting list status. Table 1 presents the sample profile.

**Table 1:** Summary of participants’ characteristics (N = 383).

Variable	Categories
Hospital N(%)	MSF Belgian Field Hospital 14(3.70%), Nasser Medical Complex 104(27.20%), Al-Rantisi Hospital 25(6.50%), Al-Shifa Hospital 139(36.30%), Al-Aqsa Hospital 101(26.40%)
Age N(%)	>55 years 139(36.30%), 18–24 years 31(8.10%), 25–34 years 57(14.90%), 35–44 years 66(17.20%), 45–54 years 90(23.50%)
Gender N(%)	Female 196(51.20%), Male 187(48.80%)
Marital Status N(%)	Widow 39(10.20%), Single 79(20.60%), Married 260(67.90%), Divorced 5(1.30%)
Education Level N(%)	Primary or less 47(12.30%), Preparatory 83(21.70%), Secondary 145(37.90%), University or higher 108(28.20%)
Monthly Family Income N(%)	1000-1500 NIS 57(14.90%), 1500–2000 NIS 24(6.30%), < 1000 NIS 269(70.20%), > 2000 NIS 33(8.60%)
Employment Status N(%)	Unemployed 300(78.30%), Retired 35(9.10%), Employed 48(12.50%)
Living Alone N(%)	No 324(84.60%), Yes 59(15.40%)
Having Children N(%)	No 182(47.50%), Yes 201(52.50%)
Number of Children (if yes) N(%)	More than two children 146(38.10%), One child 30(7.80%), Two children 25(6.50%), None 182(47.50%)
Chronic Diseases N(%)	No 91(23.80%), Yes 292(76.20%)
Hypertension (Chronic Disease) N(%)	No 115(30.00%), Yes 268(70.00%)
Diabetes (Chronic Disease) N(%)	No 291(76.00%), Yes 92(24.00%)
Heart Disease (Chronic Disease) N(%)	No 323(84.30%), Yes 60(15.70%)
Other (Chronic Disease) N(%)	No 337(88.00%), Yes 46(12.00%)
Number of Oral Medications N(%)	<5 186(48.60%),>10 28(7.30%), 5-10 169(44.10%)
Smoking N(%)	No 342(89.30%), Yes 41(10.70%)
Kidney Transplant Candidate N(%)	No 250(65.30%), Yes 133(34.70%)
Transplant Waiting List Status N(%)	Not Active 318(83.00%), Active 65(17.00%)
Dialysis Duration N(%)	1-3 years 96(25.10%), 3–5 years 89(23.20%), < 3 months 22(5.70%), < 1 year 61(15.90%), > 5 years 115(30.00%)

### War exposure and related traumatic events

Most of the patients lost their residency due to the war and reported complete destruction of their houses. Thirty-eight percent are currently living in tents, and 13% reported living with shared residency with relatives. Seventeen percent of patients were injured during the war; of them, sixty-one percent required hospitalization. Thirty-seven percent reported losing a family member due to war; of them, 30% lost more than 2 family members. Additionally, most patients lost their income due to the war. These indicators show high-intensity exposure: widespread displacement and home destruction, substantial bereavement, and major economic disruption. Table 2 presents war exposure and traumatic exposure of the participants during the war.

**Table 2:** Traumatic events after war (N = 383).

Variable	Categories
Current Residence N(%)	Displacement camp 141(36.80%), With relatives 49(12.80%), Rented home 29(7.60%), Original home 164(42.80%)
Lost Original Residence Due to War N(%)	No 130(33.90%), Yes 253(66.10%)
Type of Damage to Residence N(%)	Partial damage 142(37.10%), Total damage 205(53.50%), No damage 36(9.40%)
Injured During War N(%)	No 319(83.30%), Yes 64(16.70%)
Injury Required Hospitalization (if injured) N(%)	Injury no hospital 25(6.50%), Injury with hospital 39(10.20%), No 319(83.30%)
Lost First-Degree Relative Due to War N(%)	No 241(62.90%), Yes 142(37.10%)
Number of Lost First-Degree Relatives N(%)	More than two 43(11.20%), Two 36(9.40%), None 242(63.20%), One 62(16.20%)
Lost Main Income Source Due to War N(%)	No 99(25.80%), Yes 284(74.20%)

### Home destruction, anxiety, and PTSD prevalence

Normality of the variables was assessed visually using histograms and Q-Q plots, which showed non-normally distributed data (S1 Fig, S2 Fig, S3 Fig, S4 Fig, S5 Fig, S6 Fig), and statistically using the Shapiro-Wilk test, which shows a P value<0.05, which supports that the data are not normally distributed (S1 Table). High rates of symptomatic mental disorders were noted. The anxiety median(IQR) score was 18 (12, 26). The majority of participants suffered from moderate anxiety or higher. Regarding PTSD symptoms, 58% of participants had significant symptoms, as defined by having a PCL-5 score>33, while 42% of participants had PCL-5 scores less than or equal to 33. The median (IQR) PCL-5 score was 37(25, 50). The median home destruction score was 2 (IQR: 1–2), corresponding to partial destruction as the central tendency.

### Correlation analysis

Spearman’s rank correlation analysis shows that there was a statistically significant, very weak positive correlation between home destruction and PTSD (rho = 0.14, p = 0.01). A very weak positive correlation was found between home destruction and anxiety score (rho = 0.11, p = 0.03). Furthermore, a strong positive correlation was observed between Anxiety and PTSD (rho = 0.62, p < 0.001), suggesting the potential for mediation. Although the correlation between home destruction and PTSD is statistically significant, the magnitude (rho = 0.14) is very weak, suggesting that home destruction alone explains little variance in PTSD. This makes the strong mediation finding driven primarily by the anxiety PTSD link (rho = 0.62). Visual inspection of the scatterplots confirmed the linear nature of these relationships (S7 Fig, S8 Fig and S9 Fig). After adjusting for multiple comparisons using the holm method, only the association between PCL and (gender, Anxiety and home destruction) (Adjusted p value<0.00), the association between anxiety score and (PTSD score and home destruction) (Adjusted p value<0.00), the association between home destruction severity and (Current residency, PCL score and home destruction) (Adjusted p value<0.00) remained statistically significant (S2 Table). A total of 15 pairwise associations were included in the multiple-comparison adjustment. The Holm procedure was applied to control the family-wise error rate at α = 0.05.

### Mediation analysis

Total effect of home destruction on PTSD (c path) had a β = 3.51, SE = 1.29, z = 2.72, p = 0.01. Home destruction significantly predicted the mediator (a path: β = 2.01, SE = 0.77, z = 2.63, p = 0.01, standardized β = 0.13). When both home destruction and anxiety score were entered into the model, only anxiety score remained a significant predictor of PTSD (b path: β = 1.07, SE = 0.06, z = 16.90, p < 0.001, standardized β = 0.64), while home destruction became non-significant (c′ path: β = 1.36, SE = 1.05, z = 1.29, p = 0.20, standardized β = 0.05), indicating statistical full mediation.

**Fig 1:**
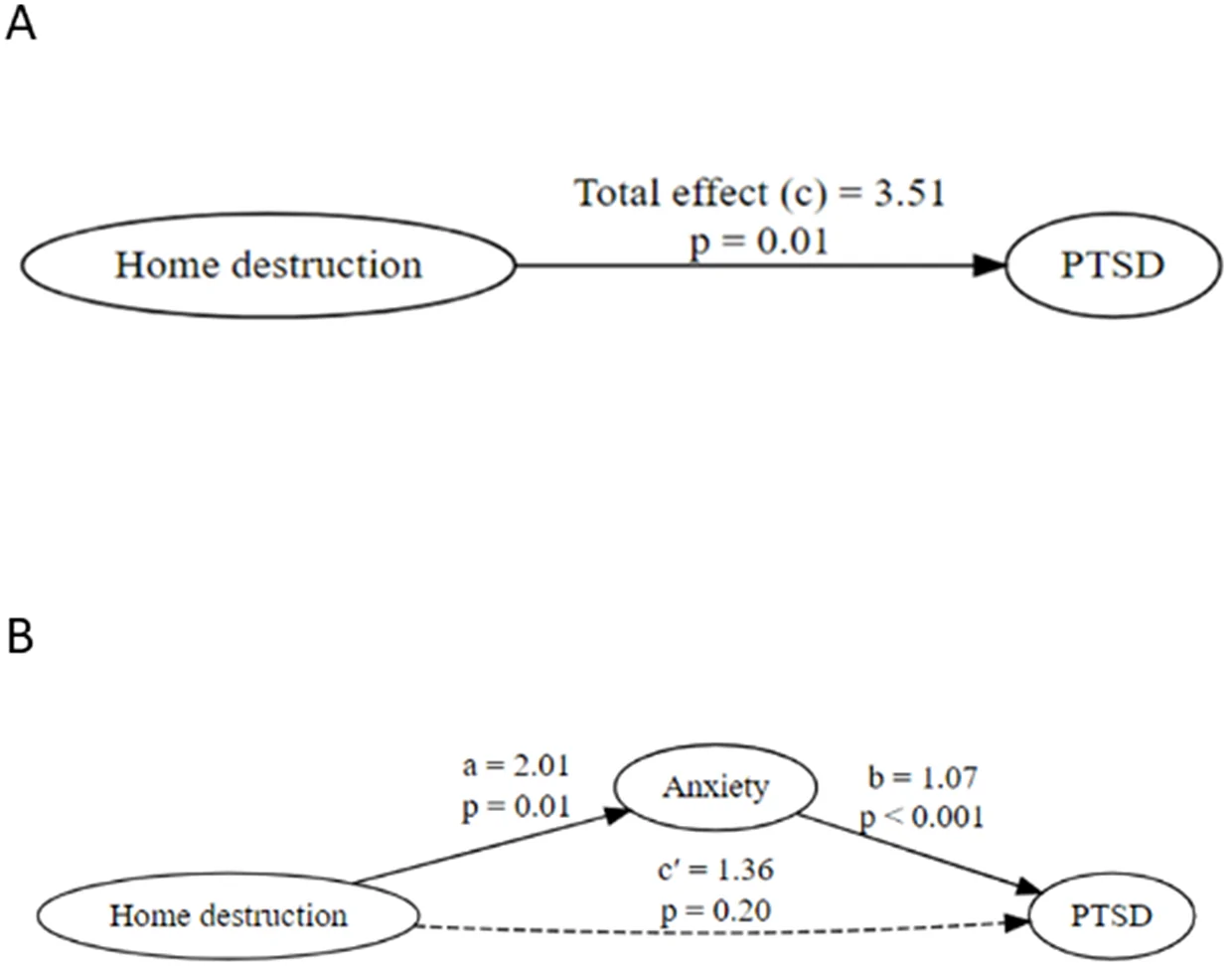
The exploratory mediation model shows anxiety as a full mediator of the relationship between home destruction and PTSD. Fig 1A shows the total effect model. Fig 1B shows the exploratory mediation model with anxiety included, demonstrating that the direct effect becomes non-significant (dashed line) while the indirect path through anxiety remains significant. Values represent unstandardized regression coefficients.

The previously significant relationship between home destruction and PTSD was completely accounted for by anxiety. The indirect effect (a × b) of home destruction on PTSD through anxiety was 2.16 (SE = 0.83). The Sobel test further supported this finding (z = 2.60, p = 0.01) (Table 3). Fig 1 illustrates the exploratory mediation model, showing both the total effect (Fig 1A) and the mediated model (Fig 1B). The model explained 2% of the variance in anxiety (R^2^ = 0.02) and 42% of the variance in PTSD score (R^2^ = 0.42).

**Table 3:** Mediation Analysis Results: Anxiety as a Mediator of the Relationship Between Home Destruction and PTSD.

Path	standardized β	SE	z	p	Unstandardized β	95% CI of the Unstandardized β
Path a: Home destruction → Anxiety	0.13	0.77	2.63	0.01	2.01	[0.48, 3.46]
Path b: Anxiety → PTSD	0.64	0.06	16.90	<0.001	1.07	[0.95, 1.20]
Path c′: Home destruction → PTSD (direct effect)	0.05	1.05	1.29	0.20	1.36	[-0.67, 3.50]
Indirect effect (a × b)	0.08	0.83	2.60	0.01	2.16	[0.51, 3.76]
Path c: Total effect	0.14	1.29	2.72	0.01	3.51	[0.99, 6.11]

### Conditional/adjusted mediation

After using the Holm method, we have included the remaining significant variables as covariates in our adjusted exploratory mediation model, including gender and current residency. The adjusted exploratory mediation analysis showed that home destruction was significantly associated with anxiety (β = 3.05, p = 0.01), and anxiety was associated with PTSD (β = 1.08, p = 0.00). The direct effect of home destruction on PTSD was not statistically significant (β = −0.71, p = 0.65). However, the indirect effect of home destruction on PTSD through anxiety remained significant (β = 3.28, 95% bootstrap CI [0.90, 5.80]). These results indicate an indirect-only mediation effect after controlling for gender and current residency (Table 4). The model explained 7% of the variance in anxiety (R^2^ = 0.07) and 43% of the variance in PTSD score R^2^ = 0.43. Because the total effect in the adjusted model was not statistically significant, the indirect-only effect should be interpreted with caution; the model primarily demonstrates that anxiety is strongly associated with PTSD, while home destruction adds little unique variance after adjusting for covariates. The regression panel of the covariates is explained in S3 Table.

**Table 4:** Adjusted Exploratory Mediation Effects of Home Destruction on PTSD via Anxiety (Controlling for Gender and Current Residency).

Effect / Path	B	SE	z	p	95% Bootstrap CI(Unstandardised)	β (Std.)
**Path a: Home destruction→ Anxiety**	3.05	1.13	2.71	0.01	[0.83, 5.27]	0.20
**Path b: Anxiety → PTSD**	1.08	0.06	16.93	0.00	[0.95, 1.20]	0.64
**Indirect effect (a × b)**	3.28	1.22	2.69	0.01	[0.90, 5.80]	0.13
**Direct effect (c**′**)**	−0.71	1.54	−0.46	0.65	[−3.80, 2.32]	−0.03
**Total effect (c)**	2.58	1.92	1.34	0.18	[−1.30, 6.30]	0.10

## Discussion

This study investigated whether anxiety mediates the association between home destruction and post-traumatic stress symptoms (PTSD) among hemodialysis patients in Gaza after October 7, 2023. In a cohort marked by severe war exposure, we found very high rates of anxiety and PTSD and a mediation pattern in which anxiety statistically accounted for the association between home destruction and PTSD – both in unadjusted and adjusted models – but causality cannot be inferred.

Our findings revealed an exceptionally high burden of psychological distress among hemodialysis patients affected by the recent conflict in Gaza. Anxiety was prevalent in 83.6% of participants at moderate or higher severity, and 58% met the clinical cutoff for PTSD (PCL-5 > 33). When compared to similar research in Gaza during the 2023–2024 conflict, the anxiety prevalence observed in our study exceeds that reported in the general population (65%) and among displaced individuals (79.3%) [2]. PTSD prevalence in our cohort (58%) is somewhat lower than the 83.5% reported in the general population and the 88.2% among displaced groups, yet remains clinically significant. These differences may reflect the unique context of dialysis care, which provides structured routines and social contact that could partially buffer PTSD severity, while persistent health-related uncertainty amplifies anxiety. However, our data were collected nearly two years after the October 2023 events, whereas Zughbur et al. assessed mental health within the first year. Differences in prevalence may reflect adaptation, survivor bias, or changes in conflict intensity. Comparable patterns have been documented in other vulnerable groups, such as dialysis patients during the COVID-19 pandemic, where death anxiety and depression affected over 60% of patients, reinforcing the notion that chronic illness magnifies psychological vulnerability during crises [5].

Correlation analysis demonstrated that home destruction was positively related to both PTSD and anxiety, while anxiety showed a strong positive correlation with PTSD. After controlling for multiple testing using the Holm method, associations among PTSD, anxiety, and home destruction remained statistically significant, alongside links with gender and current residency. The apparent discrepancy between a significant correlation (home destruction ↔ PTSD) and a non-significant direct effect in the exploratory mediation model is resolved by recognizing that correlations do not account for mechanisms. A major contribution of this study is its demonstration that anxiety functions not merely as a co-occurring symptom, but as a modifiable psychological mechanism linking structural loss to trauma-related outcomes in a medically fragile population. This extends existing conflict-related mental-health research by showing that structural war-related harms—such as home destruction—do not exert their influence uniformly across populations. Instead, they trigger psychological pathways that are amplified by clinical vulnerability and dependence on unstable healthcare systems [24]. This conceptual and empirical contribution strengthens theoretical models of trauma by illustrating how chronic threat environments interact with life-sustaining medical dependence to heighten anxiety-driven PTSD risk.

Similar dynamics have been reported in war-affected cohorts, where anxiety and depression either mediate or dominate the association between traumatic exposures and downstream psychopathology. For example, among Gazan civilians during the 2023–2024 war, depression and anxiety fully mediated the bidirectional relationship between PTSD and psychotic experiences—rendering direct effects non-significant once affective symptoms were accounted for [24]. In disaster contexts, structural losses like home destruction primarily affect health through psychological states like depressive symptoms and social mechanisms such as social cohesion, rather than through a direct pathway [20].

Our findings are particularly distinctive because hemodialysis patients constitute a population whose survival is directly shaped by the continuity of medical services. The destruction of homes for these individuals does not simply represent material loss; it undermines the daily logistics of accessing dialysis, storing medications, managing hygiene, and reaching functional healthcare centers. This amplifies uncertainty, threat perception, and fear in ways that are qualitatively different from the general population. By demonstrating that anxiety fully mediates the relationship between home destruction and PTSD specifically in dialysis patients, the study provides novel evidence that structural violence interacts with medical dependence to generate heightened psychological vulnerability. Similarly, in a large Gazan civilian sample from the same conflict, Fekih Romdhane et al. (2025) reported that depression and anxiety fully mediated the relationship between trauma exposure and psychotic experiences, reinforcing that affective symptoms are often the mechanistic pathway [5]. These findings align with the shared vulnerability framework (common risk factor theory), which posits that structural stressors increase risk for multiple mental health outcomes through a shared affective pathway [11,24].

Convergent evidence from disaster and conflict research supports such common-risk pathways: resilience and social support have mediated the relationship between disaster exposure and PTSD or depression, while depressive symptoms and diminished social cohesion have mediated the link between home loss and cognitive disability [20,25]. Likewise, unhelpful coping has functioned as a causal mediator connecting negative appraisals to PTSD and depression in longitudinal designs [26]. These studies collectively reinforce that structural adversity tends to act through modifiable psychological and social processes.

To our knowledge, no randomized trial of anxiety interventions for dialysis patients in a war zone exists. Our recommendation therefore draws on evidence from non-conflict dialysis populations and conflict affected general populations, and should be tested in feasibility studies first. By identifying anxiety as a potentially modifiable mechanism, our study also identifies a realistic intervention target. Even when structural determinants such as housing reconstruction or restoration of infrastructure cannot be quickly resolved, anxiety-focused interventions (e.g., brief CBT, psychoeducation, relaxation-based therapies) can be embedded within dialysis workflows [27,28]. This is especially important in settings where medical fragility and instability interact, and where psychological distress may hinder treatment adherence, dialysis attendance, and overall quality of life [29]. Thus, the study not only contributes theoretically but also provides actionable insights for clinical and humanitarian practice.

Adjusted mediation controlling for gender and current residency reproduced an indirect-only mediation pattern. However, because our data are cross-sectional, the temporal ordering of home destruction, anxiety, and PTSD cannot be determined. Therefore, the observed mediation should be interpreted as a statistical association rather than evidence of a causal mechanism. Nevertheless, our findings are broadly consistent with longitudinal and causal mediation studies conducted in disaster- and conflict-affected populations, where psychological responses such as anxiety, distress, maladaptive coping, and resilience have been shown to partially explain the association between traumatic exposures and subsequent mental health outcomes [20,21,25,26,30,31]. Future prospective studies following individuals over time are needed to determine whether anxiety temporally precedes PTSD symptoms and mediates the psychological impact of home destruction and displacement among medically vulnerable populations.

This study has several limitations that should be acknowledged. First, reliance on self-reported measures may introduce recall bias and social desirability effects. Second, the sample was restricted to hemodialysis patients in Gaza, which may limit generalizability to other populations or contexts. Second, potential confounders such as pre-existing psychiatric conditions, medication adherence, and socioeconomic status were not fully controlled. Third, while cross-sectional mediation analyses are common in trauma research and can illuminate potential pathways when grounded in theory, they cannot establish causality or directionality. Fourth, the DASS-21 anxiety subscale and PCL-5 share some symptomatic content, which may contribute to the strong observed correlation and the full mediation finding. This overlap is common in trauma research and suggests that anxiety symptoms captured here may partly reflect core PTSD features. Fifth, home destruction was assessed with a single item and is likely correlated with other war-related stressors. Future studies should consider composite trauma exposure indices or examine specific pathways from multiple exposures. Sixth, our sample only includes patients who survived and continued dialysis until the data collection period (August November 2025). We cannot account for patients who died, emigrated, or stopped dialysis due to the war, which may have introduced survivor bias. Seventh, data were collected by interviewers who were not blinded to the study objectives, potentially introducing interviewer bias. However, the use of validated scales with standard wording may have mitigated this risk. Eighth, the PCL 5 cutoff of >33 was derived from Western general population samples. Its diagnostic accuracy in Palestinian dialysis patients has not been validated, so PTSD prevalence estimates should be considered provisional. Additionally, the bivariate associations between home destruction and both anxiety and PTSD were small in magnitude, indicating that home destruction is one of many contributing factors in this high-trauma environment. Nevertheless, its effect operated entirely through anxiety in the statistical model, highlighting a potential pathway worthy of further investigation.

## Conclusion

In this cross-sectional study, anxiety statistically accounted for the association between home destruction and PTSD symptoms, suggesting that it may be a key psychological bridge. However, longitudinal research is needed to confirm temporal ordering. This advance understanding of trauma mechanisms in medically vulnerable populations and underscores the urgent need to integrate mental-health interventions into dialysis services within conflict settings (e.g., cognitive behavioral therapy for anxiety, relaxation training, or brief psychoeducation about worry), rather than focusing solely on trauma processing. All recommendations are based on statistical associations and should be tested in interventional or longitudinal studies before policy implementation.

## Supporting information

S1 Fig Histogram of PTSD Scores. (TIF)

S2 Fig Histogram of Anxiety Scores. (TIF)

S3 Fig Histogram of Home Destruction Scores. (TIF)

S1 Table Normality Assessment of Key Study Variables Using Shapiro–Wilk Test. (XLSX)

S4 Fig Q-Q Plot Assessing Normality of Home Destruction Scores. (TIF)

S5 Fig Q-Q Plot Assessing Normality of Anxiety Scores. (TIF)

S6 Fig Q-Q Plot Assessing Normality of PTSD Scores. (TIF)

S7 Fig Scatter Plot of Home Destruction vs. PTSD Scores. (TIF)

S8 Fig Scatter Plot of Home destruction vs. Anxiety Scores. (JPG)

S9 Fig Scatter Plot of PTSD Scores vs. Anxiety Scores. (JPG)

S2 Table P-values of One-Way ANOVA for Potential Confounders. (XLSX)

S3 Table Regression Panel of Adjusted Mediation Model Controlling for Gender and Current Residency. (XLSX)

## Abbreviations

CBT: Cognitive Behavioral Therapy
CI: Confidence Interval
DASS-21: Depression Anxiety Stress Scale (21-item)
DM: Diabetes Mellitus
DSM-5: Diagnostic and Statistical Manual of Mental Disorders, 5th Edition
HTN: Hypertension
MSF: Médecins Sans Frontières (Doctors Without Borders)
PCL-5: PTSD Checklist for DSM-5
PTSD: Post-Traumatic Stress Disorder
SD: Standard Deviation
SEM: Structural Equation Modeling
UN: United Nations
